# Critical insights: Exploring the timeliness and consistency of critical results reporting in hematology section of a specialized healthcare clinical laboratory

**DOI:** 10.12669/pjms.42.(ICON26).15709

**Published:** 2026-04

**Authors:** Sahrish Haji, Arrosa Rahim, M. Umar Saddique, Safdar Hussain

**Affiliations:** 1 Sahrish Haji, M.Phil, Multan Institute of Kidney Diseases. Indus Hospital and Health Network Multan, Pakistan; 2 Dr. Arrosa Rahim, FCPS, Multan Institute of Kidney Diseases. Indus Hospital and Health Network Multan, Pakistan; 3 M. Umar Saddique, MLT, Multan Institute of Kidney Diseases. Indus Hospital and Health Network Multan, Pakistan; 4 Safdar Hussain, MBA, Multan Institute of Kidney Diseases. Indus Hospital and Health Network Multan, Pakistan

**Keywords:** APTT, CBC, PT, RT, Result, Timeliness, critical

## Abstract

**Background & Objective:**

A critical laboratory value refers to a test result that falls within a dangerous abnormal range, indicating a potentially life-threatening condition. Delays in reporting such results can jeopardize patient well-being. Therefore, assessing the timely reporting of critical result is key factor for identifying areas of improvement and ensuring efficient dissemination of crucial information to healthcare providers. This proactive approach enhances patient care, expedites clinical decisions, and reduces morbidity and mortality. This study aimed to assess the timeliness and consistency of critical result reporting in hematology section of pathology department in a specialized healthcare facility.

**Methodology:**

A retrospective cross-sectional study was conducted at the Multan Institute of Kidney Diseases from Jan 2022 till June 2025. The sample of CBC, PT, APTT, Reticulocyte count, MP-ICT meeting sample acceptance criteria were included.

**Results:**

During the study period total 2 52, 034 million tests were requested by clinicians in the hematology section of pathology department. The requisition data shows that CBC accounts for 181750 of the total, followed by PT - 34367, APTT - 34145, Reticulocytes -1182 and ICT Malaria - 590. A total of 6,152 hematology tests yielded critical results, of which 6,130 (99.82%) were reported within the established critical reporting benchmark. Only 11 cases (0.18%) experienced delays in notification due to various influencing factors. This reflects a high compliance rate in the timely reporting of critical alerts, demonstrating exceptional operational efficiency and adherence to patient safety protocols.

**Conclusion:**

Ensuring rapid and reliable delivery of critical haematology values is essential for timely clinical intervention and improved patient outcomes. Implementation of targeted strategies such as staff training, automated alert systems, and continuous performance monitoring can significantly enhance the accuracy and efficiency of critical result management.

## INTRODUCTION

A critical laboratory value refers to a test result that falls within a dangerously abnormal range, indicating a potentially life-threatening condition, a concept first introduced in 1972 by Dr. George D. Lundberg.^1^ Critical values represent an important link between the laboratory and clinical practice. Timely communication of these alerts to the responsible caregiver can directly shape treatment decisions and improve patient outcomes, thereby enhancing safety and overall well-being. Besides physicians and nurses, patients themselves may also be notified of such results, allowing them to make informed decisions about their care and ssible interventions.^2^ One study reported that 95% of physicians regard the urgent reporting of such alerts as valuable, with nearly two-thirds of them leading to modifications in clinical decisions or treatment strategies. To maximize the effectiveness of this practice, it is important that not only physicians but also laboratory personnel and other caregivers understand the underlying pathophysiological factors associated with critical test results.^3^

The introduction of accreditation and certification programs in clinical laboratories including the College of American Pathologists (CAP), require prompt reporting of critical results. CAP particularly emphasizes the need to identify critical values and ensure their rapid communication through methods such as telephone calls or call center services.^4^ The guidelines also define threshold values for critical results; for instance, the CAP considers a hemoglobin level of ≤6 g/dl as a critical alert.^5^

At our institution, the initial list of critical laboratory values is compiled by physicians from various clinical wards. This list undergoes a multidisciplinary review process involving department heads, nursing supervisors, and hospital administration to ensure clinical relevance and prioritization. The final selection of tests and their respective cutoff thresholds are established in accordance with the guidelines provided by the College of American Pathologists (CAP). To maintain accuracy and alignment with evolving clinical needs, the list of critical values is reviewed on a biannual basis, or more frequently if required by changes in clinical practice or regulatory standards. Through this process, the laboratory’s official critical values list and standard operating procedures (SOPs) for notification are developed.^6^

According to ISO 15189, the communication of critical results is a mandatory quality practice. A study of 623 institutions found that most physicians consider it essential for patient care, with many documenting values and adjusting therapy. However, since no universal standard exists, each institution defines its own list of critical values.^7^ The Laboratory Information System (LIS) is an integrated software platform utilized across laboratory departments to streamline the processing and reporting of test results. Upon completion of sample analysis, results are automatically interfaced from the analyzer to the LIS. The Laboratory Information System (LIS) facilitates the process by automatically flagging critical results and displaying them in a dedicated alert window. These alerts remain active until the result is verified, communicated, within the defined reporting timeframe (30 minutes). Each alert is then managed by staff in accordance with the critical reporting Standard operating procedures (SOP).^8^

Once critical alerts are communicated to the primary caregiver through a Micro SIP phone call, the details are documented in the critical window of the LIS, and the results are finalized with appropriate remarks. The occurrence of critical laboratory alerts and the promptness with which they are communicated to the responsible clinical personnel are monitored as key performance indicators (KPIs) within the laboratory quality management framework. Consistency in adhering to these timelines is evaluated regularly to ensure compliance with institutional policies and accreditation standards. Performance metrics related to critical result reporting are reviewed during quality assurance meetings and are used to guide continuous improvement initiatives.^9^

In this study, we assessed the consistency and timeliness of critical value reporting in the hematology section of clinical laboratory to identify potential for improvement. The importance of critical value reporting in healthcare has grown significantly. Prompt and precise communication of critical laboratory results is crucial for informed clinical decision-making, improving patient safety, and achieving optimal treatment outcomes.

## METHODOLOGY

A retrospective cross-sectional study was conducted at the Multan Institute of Kidney Diseases from Jan 2022 till June 2025. Data was extracted from the Laboratory Information System, compiled in Excel, and analyzed using SPSS version 26. Laboratory sample of CBC, PT, APTT, Reticulocyte count, and MP-ICT meeting sample acceptance criteria within the study period were included.

### Ethical Approval:

It was obtained from Institutional Review Board IHHN_IRB_2025_08_006, IRB Approval on 09^th^ Aug, 2025.

### Inclusion criteria:

- Properly labeled, adequately filled, non-hemolyzed and samples received in adequate vacutainer were accepted.

### Exclusion criteria:

- Clotted, hemolyzed, underfilled, overfilled and mislabeled samples were excluded.

International guidelines such as ISO 15189:2022, CLSI GP47-A, and Joint Commission International (JCI) emphasize that all critical laboratory results must be promptly identified, verified, and communicated to the responsible clinician. The benchmark standard is 100% compliance, requiring that every critical result be reported within 30 minutes of verification. Communication must be properly documented, noting the time, date, and recipient of the report. Clinicians are expected to acknowledge and act on these results promptly. Regular monitoring and audits of this process are vital to ensure patient safety and adherence to international quality standards.

## RESULTS

The dataset comprised a total of 2,52,034 million tests requested by clinicians in the hematology section during the study period.

The figure shows that out of 252,034 hematology samples analyzed, 6,152 were identified as critical results, accounting for 2.44% of the total tests. This illustrates that critical results form a small but important portion of the overall laboratory workload.

The bar plot depicts the distribution of tests performed and the corresponding critical results for each laboratory parameter. Blue bars represent the total number of tests conducted, whereas red bars denote the number of critical results detected within those tests. This visual comparison facilitates assessment of testing workload and identification of parameters associated with a higher frequency of critical findings, thereby providing insight into potential areas requiring enhanced clinical attention or quality assurance review.

Bar plots show that a total of 6,152 critical results were recorded. Among these, CBC contributed the highest number of critical alerts with 5,855 cases, followed by PT with 134, APTT with 96, Reticulocytes with 23, and ICT Malaria with 33. This highlights that CBC is the most frequently requested test, as well as the most frequent source of critical findings in our section.Fig-I

**Fig.1:**
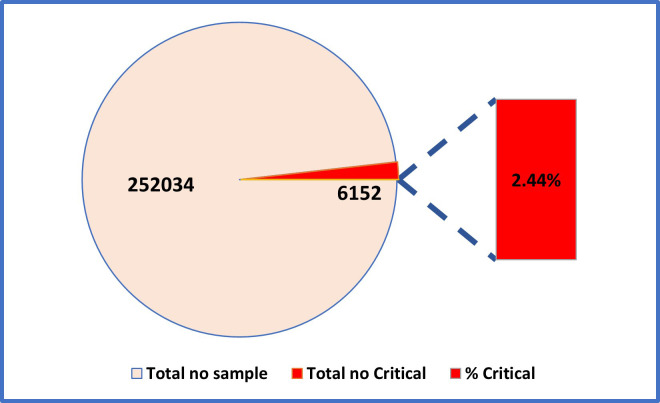
Metrics: Total Samples and Critical Results.

Out of 252,034 laboratory tests, 6,152 (2.4%) were critical and 245,882 (97.6%) were non-critical. CBC was the most commonly performed test and accounted for most of the critical results (3.2%). MP ICT showed the highest proportion of critical outcomes (5.6%) despite a smaller sample size. APTT, PT, and RETICS had relatively few critical results (0.3–1.9%). Overall, while only a small fraction of results were critical, their prompt reporting remains vital for patient safety.Fig-II

**Fig.2:**
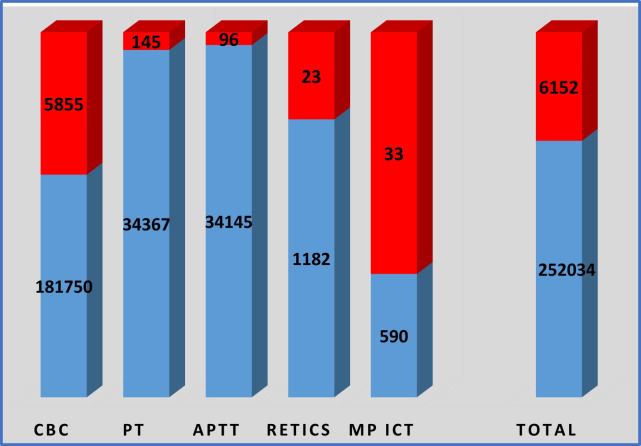
Test Requested & Critical Results.

**Fig.3:**
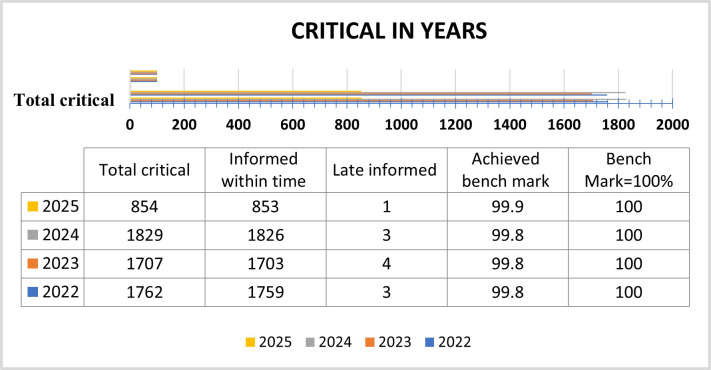
Critical Results Duration (JAN 2022- JUNE 2025)

The Chi-square analysis revealed a statistically significant association between the type of laboratory test and the occurrence of critical results (χ² = 1747.939, df = 4, p < 0.001). The p-value < 0.001 indicates a statistically significant association between test type and critical outcome. The proportion of critical results varies significantly across tests (e.g., MP ICT: 5.6%, APTT: 0.3%).

Out of the 5,855 critical alerts recorded for CBC, 2829 were related to Hemoglobin, 1761 to Total Leukocyte Count (TLC) 1265 to Platelet counts PT -145, APTT- 96 Reticulocyte count -23 and ICT MP- 33 as shown in (Table-II).

**Table-I:** Parameters and Critical Result Outcomes

Parameter * outcome Crosstabulation					
			Outcome		Total
			1	2	
Parameter	APTT	Count	96	34049	34145
		% within Parameter	0.3%	99.7%	100.0%
	CBC	Count	5855	175895	181750
		% within Parameter	3.2%	96.8%	100.0%
	MP ICT	Count	33	557	590
		% within Parameter	5.6%	94.4%	100.0%
	PT	Count	145	34222	34367
		% within Parameter	0.4%	99.6%	100.0%
	RETICS	Count	23	1159	1182
		% within Parameter	1.9%	98.1%	100.0%
Total % within Parameter		Count	6152	245882	252034
		2.4%	97.6%	100.0%	
*Chi-Square Tests*					
		*Value*	*Df*	*Asymptotic Significance(2-sided) P – value*	
Pearson Chi-Square		1747.939^a^	4	.000	
Likelihood Ratio		2411.727	4	.000	
N of Valid Cases		252034			

a. 0 cells (0.0%) have expected count less than 5. The minimum expected count is 14.40.

**Table-II:** Critical Results Parameter wise

Sr#	Test Parameter	2022	2023	2024	2025	Total
1	HB	686	754	924	465	2829
2	TLC	574	483	486	218	1761
3	PLATELET	396	394	340	135	1265
4	PT	57	34	49	5	145
5	APTT	36	18	18	24	96
6	RETCS	9	5	4	5	23
7	MP ICT	4	19	8	2	33
8	TOTAL	1762	1707	1829	854	6152

This figure represents the laboratory performance in communicating critical results within the defined turnaround time from January 2022 to June 2025. Across all four years almost all critical results were informed promptly with only 1-4 cases reported late each year. The achieved benchmark consistently remained near 99.8% - 99.9% reflecting laboratory’s strong commitment to timely reporting and adherence to quality standards.

## DISCUSSION

Most laboratories have defined critical values for at least one hematology test, with strong consensus among Ontario labs on including hemoglobin, leukocyte count, platelet count, and neutrophil count. However, thresholds vary due to differences in expert judgment, reference standards, patient populations, and analytical methods.^10^ At MIKD Laboratory, critical alerts list includes CBC parameters (HB, TLC, and PLTs), Reticulocyte count, PT, APTT and ICT Malaria. Hemoglobin: < 6.0 g/dl, WBC: < 4.0 × 10^9^/L and > 50 × 10^9^/L, Platelets: < 50 × 10^9^/L, PT > 60 seconds, INR > 5 and APTT >100 seconds. Özcan et al. conducted a pilot study analyzing 197,654 tests and found 1.02% of critical results, with a 15-minute reporting target. However, only 86.9% of alerts met this timeframe.^11^ This study evaluated critical value reporting practices across 121 institutions. On average, laboratory personnel took about four minutes to notify someone after the critical result was identified in the lab. The median time from specimen collection to notification was 56 minutes. Nearly all calls (96%) included a read-back, and 89% of critical results were initially communicated to a licensed healthcare provider.^12^ In contrast to the above studies, our laboratory achieved 99.82% success rate, with just 0.18% of alerts reported beyond 30 minutes. This difference may be due to the shorter reporting window used in the reference studies. According to CAP, a post-analytical 15-30 minutes timeframe can serve as a realistic benchmark for major critical reports. Whereas, our critical reporting SOP instructs the lab staff to report alerts of all departments, within a 30 minutes timeframe. The stringent protocol guided by CAP signifies good lab practices & quality care.^13^

The study by Vlastine et al., highlights that computerized alert systems are significantly faster than telephone calls, with far fewer delays. This efficiency is crucial in busy or resource-limited settings, where timely communication can impact patient outcomes.^13^,^14^ At our lab, critical results pop up in critical window of LIS and are thus informed to the primary caregiver via phone using Microsip. To minimize communication errors, the individual receiving the phone call is instructed to perform a read-back of the reported result to confirm accuracy. Remarks include critical parameter informed to (staff nurse /duty doctor/ clinician/) date and time. The incidence of critical alerts and the timeliness of notification to responsible staff within the target time serves as key performance indicators. A study by Paul et al., evaluated the effectiveness of critical result notification methods, comparing telephone communication (30 minutes cutoff) with computerized alerts (11-minute cutoff). The study demonstrated a higher rate of delayed notifications with telephone reporting (50.9%) compared to computerized alerts (10.9%), indicating that automated systems substantially improve timeliness and facilitate earlier clinical intervention.^12^ In our laboratory, critical alerts are efficiently managed using the SBAR tool with Micro SIP for call tracking. It logs all communications with time stamps and extension details, supporting audits and root-cause analysis. Notably, 99.82% of alerts met the 30-minute benchmark, with minor delays (0.18%) due to LIS issues, staff shortage, or recipient unavailability. A Study conducted by Howanitz et al., showed a survey result on critical result policy in which 71.4% participants lacked a policy for handling repeat critical calls, while only 20.8% of 2,301 nursing supervisors found critical value lists helpful, 94.9% of 514 physicians considered them valuable.^15^ In contrast to these, our hospital has well-defined policy for informing and reporting fresh as well as recurring critical alerts. Moreover, all doctors & nursing offices have displayed lists on their notice boards as they consider it a very effective tool.

Our section shows strong performance in critical result reporting, achieving near the 99.82% target for 30-minute notifications. Most alerts originate from abnormal CBC parameters, highlighting their clinical relevance. Despite operating in a resource-limited setting, our laboratory adheres strictly to all critical result reporting criteria, ensuring timely communication, documentation, and clinical follow-up in alignment with best practices.

### Strengths of the Study:

The study’s strengths include its focused evaluation of the hematology section, providing detailed insight into the timeliness and consistency of critical result reporting. Conducted in a specialized healthcare setting, it reflects real-world practices and has practical applicability. Its adherence to international standards ensures methodological rigor and global relevance, while quantitative analysis offers objective data to support quality improvement. Overall, the study contributes to enhancing laboratory quality management and timely communication of critical results.

### Limitations of the Study:

It includes a single center design, the findings may not be generalizable to other settings. Its focus on the hematology section limits broader applicability, and the lack of qualitative input from staff restricts insights into human and organizational factors. Additionally, uncontrolled variables such as workload, equipment issues, and clinician availability may have affected the timeliness of critical result reporting.

## CONCLUSION

Our study highlights the critical importance of prompt and efficient communication of hematology test alerts within hospital settings. Despite workload pressure and limited resources, adherence to established protocols is consistently maintained. To further enhance reporting quality, continuous training of laboratory personnel remains essential. The integration of evidence-based guidelines with advanced technologies can strengthen critical value reporting, ultimately improving patient safety, clinical decision-making, and overall quality assurance.

### Author`s contribution:

**SH:** Study concept, study design, data interpretation, manuscript writing.

**AR**: literature search and manuscript writing.

**US**: data analysis, data collection and interpretation.

**SH**: data collection. Critical Review.

All authors have read the final version and are responsible and accountable for the accuracy and integrity of the work.
